# Pharmacokinetics of Matrine in Pigs After Gavage Administration of Matrine Alone and in Combination with Amoxicillin

**DOI:** 10.3390/ani15172502

**Published:** 2025-08-25

**Authors:** Ruonan Li, Danna Zhou, Huiyu Hu, Fuhao Wang, Xiaoling Lv, Lei Sun, Xueyan Sun, Daojin Yu, Bo Yang

**Affiliations:** 1 University Key Laboratory for Integrated Chinese Traditional and Western Veterinary Medicine and Animal Healthcare in Fujian Province, Fujian Key Laboratory of Traditional Chinese Veterinary Medicine and Animal Health, College of Animal Sciences, Fujian Agriculture and Forestry University, Fuzhou 350002, China; 2 Key Laboratory of Prevention and Control Agents for Animal Bacteriosis (Ministry of Agriculture and Rural Affairs), Hubei Provincial Key Laboratory of Animal Pathogenic Microbiology, Institute of Animal Husbandry and Veterinary, Hubei Academy of Agricultural Sciences, Wuhan 430064, China; 3 Fujian Sunner Development Co., Ltd., Nanping 354100, China; 4 National Reference Laboratory of Veterinary Drug Residues, Huazhong Agricultural University, Wuhan 430070, China

**Keywords:** pharmacokinetics, matrine, amoxicillin, pig, drug–drug interaction

## Abstract

Matrine (MT) is a potential resistance reversal agent. However, little is known about its pharmacokinetics (PK) in pigs. This study aimed to investigate the PK of MT in pigs after gavage administration alone and in combination with amoxicillin (AMO). Twenty-four pigs were randomly assigned to three groups: A (MT, 50 mg/kg), B (AMO, 50 mg/kg), and C (MT + AMO, 50 mg/kg each). Blood samples were collected at predetermined time points post-administration and analyzed using liquid chromatography–tandem mass spectrometry. PK parameters were calculated using a one-compartment model. The results showed that MT was absorbed and eliminated rapidly in pigs. The maximum concentration (C_max_), time to maximum concentration (T_max_), area under the curve from 0 to 36 h (AUC_0–36 h_), apparent clearance (Cl/F), elimination rate constant (k_e_), and absorption rate constant (k_a_) for group A were 1345.55 ± 302.94 μg/L, 2.03 ± 0.14 h, 3979.10 ± 1260.85 h·μg/L, 13.72 ± 4.30 L/h/kg, 1.07 ± 0.20 h^−1^, and 0.46 ± 0.09 h^−1^, respectively, versus 2071.70 ± 715.49 μg/L, 1.27 ± 0.36 h, 9113.80 ± 3152.85 h·μg/L, 6.17 ± 2.48 L/h/kg, 2.08 ± 0.55 h^−1^, and 0.44 ± 0.24 h^−1^ for group C. AMO significantly altered the PK profiles of MT.

## Simple Summary

Matrine (MT), a quinolizidine alkaloid isolated from *Sophora* spp., has been demonstrated by previous studies to be a potential resistance reversal agent. Its use in combination with β-Lactams such as amoxicillin (AMO) may effectively treat intestinal infections caused by AMO-resistant pathogenic bacteria. The aim of this study was to investigate the pharmacokinetics (PK) of MT in pigs after gavage administration of MT alone and in combination with AMO. The results showed that MT exhibited rapid absorption and elimination in pigs. The PK profiles of both MT and AMO underwent significant alterations after their combined administration, providing evidence of the pharmacokinetic drug–drug interactions (PK-DDIs) between the two drugs. To the best of our knowledge, this study represents the first investigation into the PK profiles of MT in pigs. The results provide new insights into the disposition of MT in pigs and the PK-DDIs between MT and AMO, which will facilitate the evaluation of MT’s therapeutic efficacy in pigs.

## 1. Introduction

Bacterial intestinal infections, such as porcine colibacillosis, have caused large economic losses to the global pig industry every year [1,2]. In veterinary clinical practice, β-lactam antibiotics are commonly used drugs for the prevention and treatment of bacterial intestinal infections. Unfortunately, the increasing resistance of intestinal pathogenic bacteria to β-lactams is rendering these drugs less effective. Amoxicillin (AMO) is a typical β-lactam antibiotic. Currently, strains of *Escherichia coli* (*E. coli*) isolated from many pig farms in China are essentially resistant to it [3,4,5]. Hence, there is an urgent need to find a strategy to restore the susceptibility of intestinal pathogenic bacteria to β-lactams.

Some phytochemicals have been found to possess unique antibacterial activities, and their use alone or in combination with existing antibiotics may offer solutions to this challenge [6]. Matrine (MT) is one of the major quinolizidine alkaloids isolated from *Sophora flavescens* Ait and *Sophora alopecuroides* [7]. It exhibits a variety of biological properties such as insecticidal, antimicrobial, antiviral, anticancer, anti-inflammatory, analgesic, and antifibrotic activities [8], and may induce nephrotoxicity [9], hepatotoxicity [10], neurotoxicity [11] and reproductive toxicity [12]. In agriculture, MT is primarily applied as an insecticide, while in veterinary clinical practice, it is occasionally used for ectoparasite control (such as sarcoptic mites) in animals. Previous studies showed that MT could reverse the resistance of *E. coli* [13,14,15], *Haemophilus parasuis* [16], and *Pseudomonas aeruginosa* [17] to a variety of antibiotics including β-lactams. The combined administration of MT, berberine hydrochloride and gentamicin sulfate significantly enhanced the therapeutic efficacy against multidrug-resistant avian pathogenic *E. coli* infections [13,18]. In the mouse thigh infection model, co-administration of MT and ciprofloxacin led to a marked reduction in bacterial load at the infection site, with this effect being significantly superior to that observed in the single-drug treatment groups [14]. *Sophora alopecuroides* alkaloids, which are rich in MT, have also been confirmed to restore the susceptibility of *E. coli* to multiple antibiotics in vitro [15,18,19,20,21]. Notably, a 1024-fold reduction in the minimum inhibitory concentration (MIC) of AMO was observed in two antibiotic-resistant *E. coli* strains after treatment with *Sophora alopecuroides* alkaloid extracts [20]. Another study revealed that MT specifically inhibits the biofilm formation in antibiotic-resistant *E. coli* [22], thereby enhancing the susceptibility of these strains to antibiotics and reducing their pathogenicity to hosts. Such antibiotic resistance reversal activity makes MT a potential therapeutic or prophylactic drug for porcine bacterial intestinal infections.

Nevertheless, it remains unclear whether MT can also exert resistance reversal activity in pigs. The lack of pharmacokinetics (PK) data for MT in pigs is one of the important reasons for the inability to accurately evaluate the in vivo efficacy of MT (i.e., its resistance reversal activity in pigs). To our knowledge, the PK profiles of MT have been described in rats [23,24,25,26,27], dogs [28], rabbits [29], and humans [30]. The PK of AMO have also been investigated in pigs [31,32,33,34]. Previous studies indicate that MT is rapidly absorbed after oral administration, with a time to maximum concentration (T_max_) values all within 2.5 h [23,24,25,26,27,28,29,30], but exhibits low bioavailability, with only 17.1 ± 5.4% in rats [24]. MT exhibits widespread distribution in the body, and the apparent volume of distribution (V_d_) values are significantly larger than the average body weights [24,26]. MT is not metabolized by Cytochrome P450 enzymes or UDP-glucuronosyltransferases, and does not undergo biliary excretion [24]. As for AMO, it is absorbed rapidly after oral administration, and the mean peak plasma concentration is achieved within 2 h [31]. However, the bioavailability of AMO is relatively low (approximately 25–31%) [32]. The proportion of bound AMO is approximately 24% in porcine blood [33]. A rapid reduction in plasma AMO concentrations is observed after administration, with half-lives less than 10 h [31]. Renal excretion is the primary elimination pathway of this drug [32].

The present study aimed to characterize the pharmacokinetic (PK) profiles of MT in pigs after gavage administration of MT alone and in combination with AMO. Additionally, the potential pharmacokinetic drug–drug interactions (PK-DDIs) between MT and AMO were also investigated.

## 2. Materials and Methods

### 2.1. Materials

The standards of MT (CAS No. 519-02-8, white crystalline powder, purity ≥ 98% by HPLC) and AMO (CAS No. 61336-70-7, amoxicillin trihydrate, purity ≥ 98% by HPLC) were purchased from Shanghai Yuanye Biotechnology Co., Ltd. (Shanghai, China) and Dr. Ehrenstorfer GmbH (Augsburg, Germany), respectively. The standard stock solutions (1000 μg/mL) of MT and AMO for sample analysis were prepared separately in acetonitrile and stored at –20 °C. The working standard solutions (100 μg/mL) of each compound were prepared weekly by diluting the stock standard solutions in acetonitrile and stored at 4 °C. LC–MS grade acetonitrile and formic acid were purchased from Merck KGaA (Darmstadt, Germany). Deionized water (18.25 MΩ·cm) produced by a Milli-Q system (Millipore Co., Bedford, MA, USA) was used throughout the study. AMO soluble powder (30%) for the animal experiment was purchased from Wuhan Hvsen Biotechnology Co., Ltd. (Wuhan, China).

### 2.2. Animals

Twenty-four healthy six-week-old crossbred (Landrace × Large White) pigs (12 male and 12 female), weighing 10.66 ± 0.67 kg, were purchased from Fujian Minlv Three Dimensional Agricultural Comprehensive Development Co., Ltd. (Ningde, China). The pigs were randomly assigned to three treatments with eight replicates per treatment, namely group A (MT, 50 mg/kg); group B (AMO, 50 mg/kg); and group C (MT, 50 mg/kg + AMO, 50 mg/kg). The sample size (8 individuals per group) was determined in accordance with Announcement No. 1247 of the Ministry of Agriculture of the People’s Republic of China [35]. The dosages of MT and AMO (50 mg/kg) were determined based on previous studies [15,23,24,25,26,27,28,29,30,34,36] and our pre-experiment, considering their efficacy against *E. coli*, as well as the safety of their use in pigs and food safety. The three groups were raised separately in three pig beds to acclimatize for seven days prior to the experiment. The environment temperature and humidity were maintained at 21 ± 6 °C and 72–88%, respectively, and good ventilation was ensured. During the experiment, the pigs were provided with drug-free feed and water. All animal experiments were performed in accordance with the Guide for the Care and Use of Laboratory Animals [37] and were approved by the Research Ethics Committee of the College of Animal Science, Fujian Agriculture and Forestry University (No. PZCASFAFU21031).

### 2.3. Analytical Method

The concentrations of MT and AMO in porcine plasma samples were determined using liquid chromatography–tandem mass spectrometry (LC–MS/MS) methods. Briefly, 500 μL of plasma sample was deproteinized with an equal volume of acetonitrile. After vortexing and centrifuging at 10,000 rpm for 5 min, the supernatant was transferred to a clean centrifuge tube. For the determination of MT, the supernatant was filtered through 0.22-μm PTFE syringe filters (Lizhu Biological Technology Co., Guangzhou, China) prior to analysis. For the determination of AMO, the supernatant was diluted two-fold with deionized water, and then filtered through 0.22-μm PTFE syringe filters for LC–MS/MS analysis.

Samples that contained MT were analyzed using an Agilent 6460 Triple Quadrupole LC–MS system (Agilent Technologies Inc., Santa Clara, CA, USA). Chromatographic separation was performed at 35 °C using a ChromCore C_18_ column (2.1 mm × 100 mm, 3 μm) from NanoChrom Technologies (Suzhou) Co., Ltd. (Suzhou, China). The mobile phases were (A) 1% formic acid in water and (B) acetonitrile, and the corresponding gradient profile was 80% A for 0.2 min, then a linear gradient to 50% A at 2 min and returned to 80% A in 0.5 min. The flow rate was 0.2 mL/min, and the injection volume was 2 μL. The mass spectrometer was operated in positive electrospray ionization (ESI+) mode with multiple reaction monitoring (MRM) resolution. Nitrogen was used as the desolvation gas at a flow rate of 720 L/h. Other parameters were listed in Table S1.

Sample that contained AMO were analyzed using a Waters UPLC-Xevo TQ-S Micro system (Waters, Milford, MA, USA). Chromatographic separation was performed at 35 °C on a Waters ACQUITY UPLC BEH C_18_ column (2.1 mm × 100 mm, 1.7 μm; Waters, Milford, MA, USA). The mobile phase composition was identical to that for the analysis of MT, and the corresponding gradient profile was 98% A for 0.2 min, then a linear gradient of 20% A at 3.5 min, and then it returned to 80% A in 1.5 min. The flow rate was 0.3 mL/min, and the injection volume was 3 μL. The MS was operated in ESI+ with MRM resolution. The desolvation gas (N_2_) flow was 800 L/h. Other parameters were listed in Table S1.

The analytical performances of the two LC–MS/MS methods were evaluated according to USFDA guidelines [38]. Five batches of blank porcine plasma samples from different sources and twenty-five blank porcine plasma samples spiked with MT (or AMO) at LLOQ were analyzed as mentioned above. The MRM chromatograms of blank samples and analyte-spiked blank samples were compared to evaluate the analytical specificity. Matrix effect was investigated as described in a previous study [39]. Three different concentrations of analytes (1, 25, 100 μg/L) were investigated by analyzing five replicates for each concentration. Matrix-matched calibration curves were established by spiking blank plasma extracts with known concentrations of MT or AMO. These curves were used to calculate the concentrations of MT and AMO in porcine plasma samples. The linearity of the matrix-matched calibration curves was represented by the correlation coefficient, and a correlation coefficient greater than 0.98 was acceptable. Three consecutive analytical batches including three concentrations of quality control (QC) samples (spiked at 5, 100 and 500 μg/L) with six replicates for each were performed to evaluate the analytical precision and accuracy. The relative standard deviation (RSD) and relative error (RE) were used as indicators for the evaluation of analytical precision and accuracy, respectively. The blank samples spiked with MT (or AMO) at 1, 5, 25, and 100 μg/L, with six replicates for each, were analyzed as mentioned above. The LLOQ, defined as the lowest concentration with RSD and RE ≤ 20% was used as the indicator for the evaluation of analytical sensitivity. The stabilities of MT and AMO in porcine plasma were evaluated under various conditions. The analyte-spiked blank samples at 5, 100, 500 μg/L were subjected to short-term condition (room temperature, 24 h), to long-term condition (–20 °C, seven days), and to three cycles of freeze–thaw stability studies (freezing at –20 °C for 24 h then thawing at room temperature for 12 h). Then, these samples were analyzed as mentioned above.

### 2.4. Experimental Design

The drug preparations for gavage included three aqueous solutions: MT (25 mg/mL, measured as MT), AMO (25 mg/mL, measured as AMO), and MT–AMO mixture (25 mg/mL MT + 25 mg/mL AMO). These were prepared by dissolving appropriate amounts of MT standard, AMO soluble powder, and their mixture in sterile water, respectively. The experimental design for PK study was shown in Table S2. During the experiment, the pigs in group A, group B, and group C were fasted for 12 h before drug administration. They were then administered with MT aqueous solution (50 mg/kg, measured as MT), AMO aqueous solution (50 mg/kg, measured as AMO), or MT–AMO mixture aqueous solution (50 mg/kg, measured as MT and AMO) in appropriate volumes via gavage using a gastric tube, respectively. The gastric tube was then rinsed once with 10 mL of sterile water, and the rinsing solution was completely flushed into their stomachs. Blood (approximately 3 mL) was collected via the anterior vena cava at 0 (pre-dosing), 0.25, 0.5, 1, 2, 3, 4, 5, 8, 12, 16, 24, and 36 h post-dosing. These samples were put into heparinized vacuum blood collection tubes (Blue Sail Medical Co., Ltd., Zibo, Shandong province, China) and centrifuged at 1500 rpm for 5 min to prepare plasma. The plasma samples were stored at –20 °C until analysis.

### 2.5. Data Analysis

The PK parameters for each subject were calculated using WinNonlin version 5.2.1 (Pharsight Co., Mountain View, CA, USA) through a one-compartment model. These parameters were as follows: the maximum concentration (C_max_), time to maximum concentration (T_max_), area under the curve from time 0 to 36 h (AUC_0–36h_), apparent clearance (Cl/F), elimination rate constant (k_e_), and absorption rate constant (k_a_). The plasma drug concentrations, PK parameters, and other data involved in this study were presented as means ± standard deviations (SD). The normality of these PK parameters was assessed using the Shapiro–Wilk test. The differences in C_max_, AUC_0–36h_, Cl/F, k_e_, and k_a_ between the treatment groups were analyzed using one-way ANOVA with Bonferroni *t*-test when these parameters showed homogeneous variance and normal distribution. The Kruskal–Wallis test was used for cases of heterogeneous variance or non-normal distribution. For the comparison of T_max_ between the treatment groups, the Kruskal–Wallis test was used. All statistical analyses were performed using SPSS version 21 (IBM Co., Armonk, NY, USA), and a *p*-value of <0.05 was considered significant.

## 3. Results

### 3.1. Method Validation

**Figure 1:**
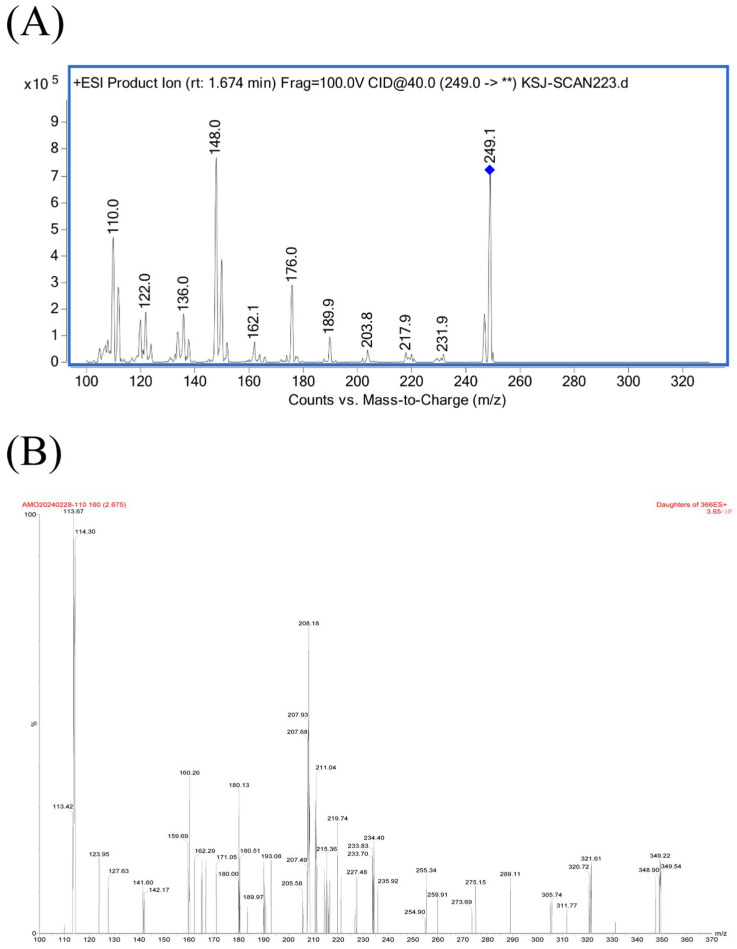
Product ion spectra of: protonated (**A**) MT and (**B**) AMO. ** denotes a fragment ion whose identity is temporarily unknown.

**Figure 2:**
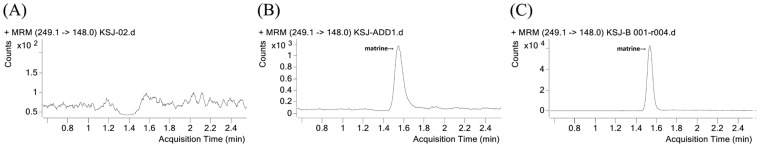
Representative MRM chromatograms of MT in porcine plasma samples. (**A**) blank plasma sample; (**B**) blank plasma sample spiked with MT at 5 μg/L (LLOQ); (**C**) plasma sample collected from a pig 8 h after a single intragastric administration of MT (50 mg/kg).

**Figure 3:**
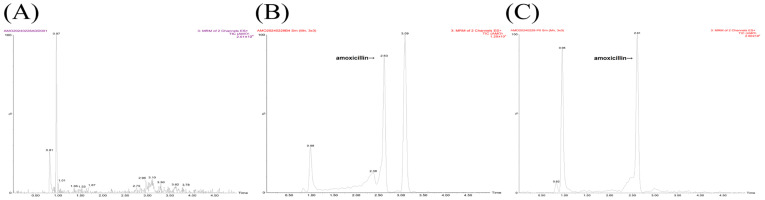
Representative MRM chromatograms of AMO in porcine plasma samples. (**A**) blank plasma sample; (**B**) blank plasma sample spiked with AMO at 5 μg/L (LLOQ); (**C**) plasma sample collected from a pig 8 h after a single intragastric administration of AMO (50 mg/kg).

As shown in Figure 1, the qualitative ions were *m*/*z* 249.1→176.0 and *m*/*z* 249.1→148.0 for MT, and *m*/*z* 366.0→208.1 and *m*/*z* 366.0→114.3 for AMO, respectively; the quantification ions were *m*/*z* 249.1→148.0 for MT and *m*/*z* 366.0→114.2 for AMO, respectively. Representative MRM chromatograms obtained from blank plasma samples, blank plasma samples spiked with MT or AMO, and plasma samples after administration of MT or AMO are presented in Figure 2 and Figure 3. The retention times of MT and AMO were approximately 1.558 and 2.630 min, respectively. There were no endogenous interfering peaks observed at the retention times of MT and AMO. The matrix effect was evaluated using the matrix factor, which was defined as the ratio of the peak area in spiked blank plasma extracts to that in water. For the analytes, the matrix factor at concentrations of 1, 25, and 100 μg/L was: MT 13.85 ± 1.26%, 12.52 ± 2.14%, and 12.82 ± 1.88%; AMO 40.21 ± 3.21%, 50.02 ± 1.70%, and 54.10 ± 2.82%, respectively. There were significant matrix suppression effects in porcine plasma samples. The specific suppression percentages of MT and AMO at 1, 25, and 100 μg/L were 86.15%, 87.48%, 87.18%, and 59.79%, 49.98%, 45.90%, respectively. The matrix-matched calibration curves showed good linearity (MT: y = 4216.10x − 8298.30, r = 0.9990; AMO: y = 62.817x + 129.90, r = 0.9949) over the concentration range from 1 to 100 μg/L. The accuracy and precision of MT and AMO are listed in Table 1. The accuracy ranged from –18.59 to 14.37% for intra-day determination and −15.18 to 13.54% for inter-day determination, respectively. The corresponding precision ranged from 1.99 to 7.87% and 0.71 to 7.62%, respectively. The developed LC–MS/MS methods showed high sensitivity in determining MT and AMO with LLOQ of 5 μg/L. The results of stability evaluation are listed in Table S3. Good stability was observed for MT and AMO under the conditions mentioned above.

### 3.2. PK of MT and AMO in Pigs

**Figure 4:**
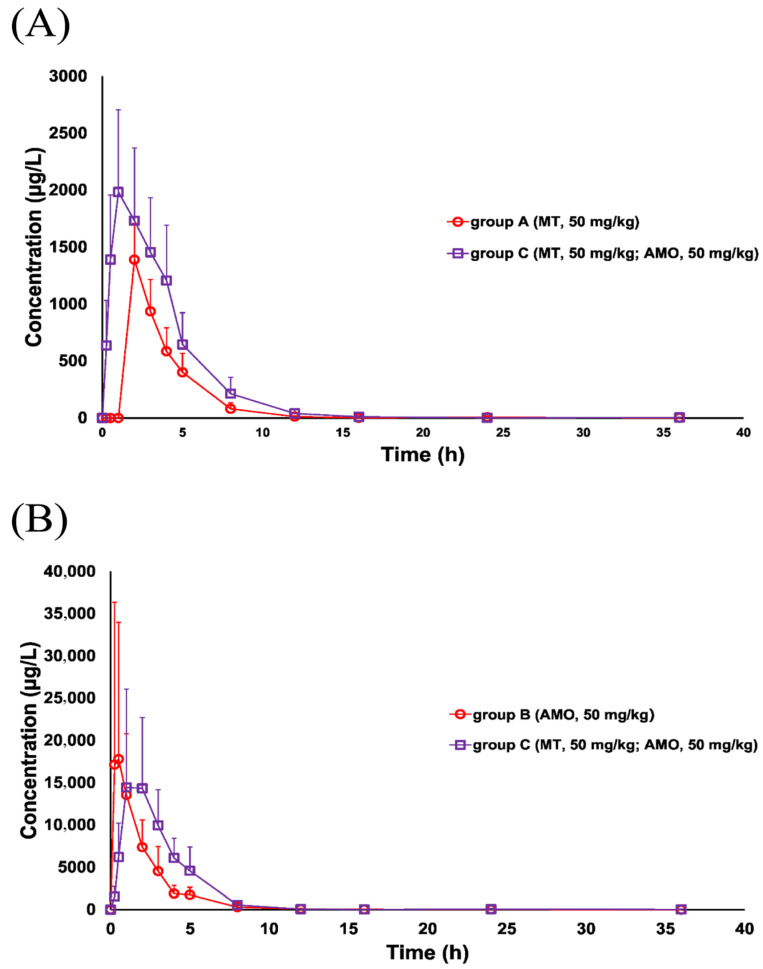
Plasma concentration–time profiles of MT and AMO in pigs (8 individuals per time point) after gavage administration of each drug alone or in combination. (**A**) MT concentrations in Group A (MT alone) versus Group C (MT–AMO combination); (**B**) AMO concentrations in Group B (AMO alone) versus Group C (MT–AMO combination).

The time courses of MT and AMO in porcine plasma are shown in Figure 4, and the main PK parameters were summarized in Table 2 and Table 3. The results showed that MT was absorbed and eliminated rapidly in pigs. After a single gavage administration, the plasma concentration of MT reached its peak within 2.21 h, and was below the LLOQ 16 h after post-administration. Compared with single MT administration, the combined use of MT and AMO resulted in a significant increase in the C_max_ (1345.55 ± 302.94 vs. 2071.70 ± 715.49, *p* < 0.05), AUC_0–36h_ (3979.10 ± 1260.85 vs. 9113.80 ± 3152.85, *p* < 0.01), and k_e_ (1.07 ± 0.20 vs. 2.08 ± 0.55, *p* < 0.01), while a significant decrease in the T_max_ (1.27 ± 0.36 vs. 2.03 ± 0.14, *p* < 0.01) and Cl/F (13.72 ± 4.30 vs. 6.17 ± 2.48, *p* < 0.01). No significant differences in the k_a_ were found between the two treatments (*p* > 0.05). For AMO, co-administration with MT significantly increased the T_max_ (0.88 ± 0.45 vs. 1.55 ± 0.36, *p* < 0.01), and k_a_ (0.33 ± 0.19 vs. 0.76 ± 0.30, *p* < 0.01) when compared with single AMO administration. No significant differences in the C_max_, AUC_0–36h_, Cl/F, and k_e_ were found between the two treatments (*p* > 0.05).

## 4. Discussion

The increasing resistance of intestinal pathogenic bacteria to β-lactams such as AMO poses significant challenges to the treatment of bacterial intestinal infections. MT, a potential resistance reversal agent, shows potential in addressing this challenge. In this study, the PK of MT was investigated in pigs after gavage administration of MT alone and in combination with AMO for the first time. These results are expected to provide new insights into the disposition of MT in pigs and the PK-DDIs between MT and AMO, which will facilitate the evaluation of MT’s therapeutic efficacy in pigs.

All concentration–time data were obtained from animal experiments based on two reliable LC–MS/MS methods. Critical analytical performance parameters such as specificity, linearity, accuracy, precision, sensitivity, and stability were well demonstrated by validation data herein. Unlike previous studies [24,26,30,40,41], notable matrix suppression effects were detected during the analysis of porcine plasma samples. Matrix-matched calibration curves were thus employed to mitigate matrix effects and ensure accurate quantification. Satisfactory linearity (r ≥ 0.999) was achieved over the concentration range of 1–100 μg/L. Plasma samples with analyte concentrations exceeding 100 μg/L (corresponding to the data at 500 μg/L concentration presented in Table 1) were diluted with blank plasma extracts before analysis, ensuring they fell within the linear range (1–100 μg/L) of the matrix-matched standard curves. The accuracy and precision of the dilution process were validated to ensure reliable quantification of these high-concentration samples.

After a single gavage administration, rapid absorption and elimination of MT were observed in pigs. The absorption rate (characterized by T_max_) was comparable to that in rats [26]. The absorption extent (characterized by C_max_ and AUC_0–36h_) was substantially lower than that in both rats and dogs [23,26,28], while the elimination (characterized by CL/F and k_e_) was substantially faster than that in rats [26]. Yang et al. found that the oral bioavailability of MT in rats was extremely low (17.1 ± 5.4%) [24]. The lower absorption extent and faster elimination observed in this study may lead to lower oral bioavailability of MT in pigs than in rats. One reason for the relatively low C_max_ and AUC_0–36h_ may be that most of administered MT dose remained unabsorbed in porcine intestinal tract. Since the intestinal tract is the primary site of intestinal pathogenic bacteria colonization in pigs, the high concentration of MT herein ensures its in vivo efficacy (i.e., its resistance reversal activity in pigs). However, the poor oral bioavailability of MT in pigs may also result in suboptimal efficacy when treating systemic infections. Formulation optimization studies are therefore warranted to enhance its oral absorption and prolong its elimination half-life. Another possible explanation may be that MT is extensively distributed in the body and accumulated in specific organs after administration. This speculation was supported by the fact that the apparent volume of distribution (V_d_) of MT in rats (2.43 ± 1.67 L/kg) was more than twice the volume of the whole body for a 0.25–0.30 kg rat [24,26], since according to PK theory, a V_d_ exceeding the animal’s body volume often indicates extensive drug distribution or drug accumulation in specific tissues or organs [42,43]. Our previous study also confirmed that MT accumulated in porcine muscle, liver, and kidney following oral administration [36]. Nevertheless, it showed no persistent tissue residue in pigs and could be accepted as a safe anti-infective agent [36]. For AMO, comparable T_max_ and Cl/F, as well as higher C_max_ and AUC, were observed in this study compared to the report by Burch and Sperling [31]. This finding is attributed to the dose administered, and may also be influenced by animal-specific factors, such as body weight and interindividual variability.

The PK profiles of MT and AMO changed significantly after the combined administration of these two drugs, indicating the occurrence of PK-DDIs between MT and AMO. As mentioned earlier, co-administered AMO significantly increased MT’s C_max_ and AUC_0–36h_. The underlying mechanism may be that co-administered AMO inhibits the uptake of MT by specific organs. Previous studies have demonstrated that certain organs, such as the kidney and liver, exhibited a strong affinity for MT [25,36]. MT tends to accumulate in these organs [36], which results in reduced concentrations in the bloodstream and consequently leads to relatively low C_max_ and AUC_0–36h_. In this study, co-administration with AMO may suppress such organ-specific uptake of MT, thereby increasing its retention in the systemic circulation and contributing to the elevated C_max_ and AUC_0–36h_. This is supported by non-compartmental analysis of our plasma MT concentration–time data, which showed that the apparent volume of distribution normalized by bioavailability (V/F) was significantly lower in the MT–AMO co-administration group than in the MT alone group (30.31 ± 15.31 L/kg versus 63.08 ± 22.24 L/kg, *p* < 0.05). Additionally, co-administered AMO significantly decreased MT’s Cl/F too. As shown in Table 2, the Cl/F was significantly decreased from 13.72 ± 4.30 L/h/kg to 6.17 ± 2.48 L/h/kg (*p* < 0.01). We hypothesize that this phenomenon may be attributed to the mutual interference of MT and AMO with each other’s renal excretion mechanisms, particularly tubular secretion. Further PK investigations are warranted to validate this hypothesis. In the case of AMO, its co-administration with MT led to a significant increase in T_max_ and k_a_, whereas no notable changes were observed in other PK parameters. This apparently “paradoxical” change may precisely reflect the complexity of AMO’s absorption process after co-administration. While the specific mechanisms remain to be elucidated, potential explanations could involve interactions with MT in the absorption process, such as modulation of oligopeptide transporter PepT1 (a key transporter for intestinal absorption of β-lactam antibiotics like AMO [44,45]) in porcine intestinal epithelial cells and alterations in gastric pH affecting AMO’s solubility. As shown in Figure 4B, co-administration with MT appears to increase the percentage of time that free concentrations of AMO remain above its MIC against *E. coli* (4 μg/mL, as reported in the study by Decundo et al. [34]) (%T > MIC). This heightened %T > MIC is particularly relevant for time-dependent antibiotics like AMO, as it directly improves therapeutic outcomes against porcine colibacillosis. Notably, the incompatibility of drugs is closely associated with PK-DDI, as physicochemical conflicts in vitro may alter drug absorption, distribution, metabolism, or excretion in vivo. Taking this into account, the physicochemical incompatibility between MT and AMO was evaluated by monitoring the stability of their mixture in equal proportions under specific conditions. Briefly, MT aqueous solutions at three concentrations (10, 0.1, 0.001 mg/mL, expressed as MT) were mixed in equal volumes with AMO solutions at corresponding concentrations (10, 0.1, 0.001 mg/mL, expressed as AMO), yielding three MT–AMO combinations at final concentrations of 5, 0.05, 0.0005 mg/mL. Five replicate samples were prepared for each combination concentration. The MT–AMO combinations were placed at room temperature (28 °C) for 2 h, followed by LC–MS/MS analysis. The recovery of both drugs ranged from 90.32% to 100.24%, indicating that the MT–AMO combinations remained stable under the above-mentioned conditions and that no evidence of incompatibility was found between MT and AMO.

## 5. Conclusions

MT exhibits rapid absorption and elimination in pigs. Additionally, the PK profiles of both MT and AMO undergo significant alterations after their combined administration, providing evidence of a PK-DDI between the two drugs. To the best of our knowledge, this study represents the first investigation into the PK profiles of MT in pigs. The results provide new insights into the disposition of MT in pigs and the PK-DDIs between MT and AMO, which will facilitate the evaluation of MT’s therapeutic efficacy in pigs.

## Figures and Tables

**Table 1:** Precision and accuracy of the LC–MS/MS methods for the determination of MT and AMO in pig plasma samples.

Analyte	Nominal Concentration (μg/L)	Intra-Day (n = 6)			Inter-Day (n = 18)		
		Determined Concentration (μg/L)	RSD (%)	RE (%)	Determined Concentration (μg/L)	RSD (%)	RE (%)
MT	5	4.13 ± 0.19	4.60	−17.33	4.24 ± 0.22	5.16	−15.18
		4.32 ± 0.28	6.46	−13.55			
		4.27 ± 0.16	3.77	−14.65			
	100	96.40 ± 3.59	3.73	−3.60	96.27 ± 3.23	3.35	−3.73
		96.49 ± 3.45	3.58	−3.51			
		95.90 ± 3.21	3.35	−4.10			
	500	537.28 ± 12.42	2.31	7.46	542.94 ± 11.99	2.21	8.59
		549.67 ± 11.11	2.02	9.94			
		541.85 ± 10.80	1.99	8.22			
AMO	5	4.74 ± 0.23	4.82	–5.19	4.40 ± 0.34	7.62	–12.07
		4.38 ± 0.34	7.87	–12.43			
		4.07 ± 0.25	6.07	–18.59			
	100	113.82 ± 4.33	3.80	13.83	112.67 ± 1.64	1.45	12.67
		113.38 ± 2.61	2.30	13.38			
		110.80 ± 3.16	2.85	10.80			
	500	571.83 ± 22.29	3.90	14.37	567.68 ± 4.01	0.71	13.54
		567.38 ± 28.79	5.07	13.48			
		563.83 ± 28.68	5.09	12.77			

**Table 2:** The PK parameters of MT in pigs after gavage administration of MT alone (group A, n = 8) and in combination with AMO (group C, n = 8).

Parameter	Unit	Group A	Group C
C_max_	μg/L	1345.55 ± 302.94 *	2071.70 ± 715.49 *
T_max_	h	2.03 ± 0.14 **	1.27 ± 0.36 **
AUC_0→36h_	h·μg/L	3979.10 ± 1260.85 **	9113.8 ± 3152.85 **
Cl/F	L/h/kg	13.72 ± 4.30 **	6.17 ± 2.48 **
k_e_	h^−1^	1.07 ± 0.20 **	2.08 ± 0.55 **
k_a_	h^−1^	0.46 ± 0.09	0.44 ± 0.24

C_max_ maximum concentration, T_max_ time to maximum concentration, AUC_0→36h_ area under the curve from time 0 to 36 h, Cl/F apparent clearance, k_e_ elimination rate constant, k_a_ absorption rate constant. * Significant difference (*p* < 0.05), ** Highly significant difference (*p* < 0.01).

**Table 3:** The PK parameters of AMO in pigs after gavage administration of AMO alone (group B, n = 8) and in combination with MT (group C, n = 8).

**Parameter**	**Unit**	**Group** **B**	**Group C**
C_max_	μg/L	14,210.40 ± 11,048.73	15,636.55 ± 8613.34
T_max_	h	0.88 ± 0.45 **	1.55 ± 0.36 **
AUC_0→36h_	h·μg/L	43,167.11 ± 37,871.42	55,057.22 ± 21,125.22
Cl/F	L/h/kg	1.91 ± 1.03	1.02 ± 0.32
k_e_	h^−1^	1.44 ± 1.16	1.25 ± 0.67
k_a_	h^−1^	0.33 ± 0.19 **	0.76 ± 0.30 **

C_max_ maximum concentration, T_max_ time to maximum concentration, AUC_0→36h_ area under the curve from time 0 to 36 h, Cl/F apparent clearance, k_e_ elimination rate constant, k_a_ absorption rate constant. ** Highly significant difference (*p* < 0.01).

## Data Availability

The original contributions presented in the study are included in the article/Supplementary Materials; further inquiries can be directed to the corresponding author.

## Supplementary Materials

The following supporting information can be downloaded at: https://www.mdpi.com/article/10.3390/ani15172502/s1, Table S1: The optimized MS/MS parameters for MT and AMO; Table S2: The experimental design for PK study; Table S3: Storage stability of MT and AMO in pig plasma samples under various conditions (n = 5).

## Abbreviations

The following abbreviations are used in this manuscript:

MT	Matrine
AMO	Amoxicillin
PK	Pharmacokinetic
LC–MS/MS	Liquid chromatography–tandem mass spectrometry
C_max_	The maximum concentration
T_max_	The time to maximum concentration
AUC_0–36 h_	The area under the curve from 0 to 36 h
Cl/F	The apparent clearance
k_e_	The elimination rate constant
k_a_	The absorption rate constant
V_d_	The apparent volume of distribution
*E. coli*	Escherichia coli
MIC	The minimum inhibitory concentration
PK-DDIs	Pharmacokinetic drug–drug interactions
MS	Mass spectrometer
MRM	Multiple reaction monitoring
QC	Quality control
RSD	Relative standard deviation
RE	Relative error
LLOQ	Lower limit of quantification
SD	Standard deviation
