# Supplementary material for: Pharmacokinetics of Matrine in Pigs After Gavage Administration of Matrine Alone and in Combination with Amoxicillin
Source: Animals (Basel). 2025 Aug 25;15(17):2502. doi: 10.3390/ani15172502 (PMC12427342; doi:10.3390/ani15172502)
Supplement: Supplementary file 1 [file animals-15-02502-s001.zip › animals-3797464-supplementary.pdf]

Supplementary

Table S1. The optimized MS/MS parameters for MT and AMO.

Analyte	Qualitative ions (m/z)	Quantification ions (m/z)	Dwell time (s)	Cone voltage (V)	Collision energy (V)	Capillary voltage (Kv)	Source temperature (°C)	Desolvation temperature (°C)
MT	249.1→176.0	249.1→148.0	0.1	100	35	3.0	350	350
	249.1→148.0		0.1	100	33			
AMO	366.0→208.1	366.0→114.2	0.036	36	26	3.0	150	400
	366.0→114.2		0.036	36	12			

Table S2. The experimental design for PK study.

Groups	Total number of animals	Drug preparations	Dosage regimen
A	8	MT aqueous solution (25 mg/mL, measured as MT),	Single-dose administration via gavage at 50 mg/kg (measured as MT)
B	8	AMO aqueous solution (25 mg/mL, measured as AMO)	Single-dose administration via gavage at 50 mg/kg (measured as AMO)
C	8	MT-AMO mixture aqueous solution (25 mg/mL, measured as MT, and 25 mg/mL, measured as AMO)	Single-dose administration via gavage at 50 mg/kg (measured as MT and AMO)

Table S3. Storage stability of MT and AMO in pig plasma samples under various conditions (n = 5).

Condition	Nominal concentration (μg/L)	MT			AMO		
		Determined concentration (μg/L)	RSD (%)	RE (%)	Determined concentration (μg/L)	RSD (%)	RE (%)
−20 °C, 7 days	5	4.39 ± 0.40	9.21	−12.24	4.57 ± 0.27	5.93	−8.52
	100	89.23 ± 4.07	4.56	−10.77	93.89 ± 6.84	7.28	−6.11
	500	470.39 ± 30.46	6.48	−5.92	540.48 ± 25.61	4.74	8.10
Room temperature, 24 h	5	4.69 ± 0.40	8.45	−6.28	4.76 ± 0.37	7.83	−4.72
	100	92.07 ± 6.16	6.69	−7.93	107.59 ± 7.90	7.34	7.59
	500	479.25 ± 36.63	7.64	−4.15	553.42 ±30.84	5.57	10.68
Three freeze/thaw cycles	5	4.65 ± 0.44	9.49	−7.08	4.63 ± 0.59	12.74	−7.40
	100	89.11 ± 5.72	6.42	−10.89	93.44 ± 1.44	1.54	−6.56
	500	458.97 ± 28.88	6.29	−8.21	487.27 ± 40.65	8.34	−2.55

[a]: Mean ± SD
